# Bipyramid-templated synthesis of monodisperse anisotropic gold nanocrystals

**DOI:** 10.1038/ncomms8571

**Published:** 2015-06-26

**Authors:** Jung-Hoon Lee, Kyle J. Gibson, Gang Chen, Yossi Weizmann

**Affiliations:** 1 Department of Chemistry, The University of Chicago, 929 East 57th Street, Chicago, Illinois 60637, USA

## Abstract

Much of the interest in noble metal nanoparticles is due to their plasmonic resonance responses and local field enhancement, both of which can be tuned through the size and shape of the particles. However, both properties suffer from the loss of monodispersity that is frequently associated with various morphologies of nanoparticles. Here we show a method to generate diverse and monodisperse anisotropic gold nanoparticle shapes with various tip geometries as well as highly tunable size augmentations through either oxidative etching or seed-mediated growth of purified, monodisperse gold bipyramids. The conditions employed in the etching and growth processes also offer valuable insights into the growth mechanism difficult to realize with other gold nanostructures. The high-index facets and more complicated structure of the bipyramid lead to a wider variety of intriguing regrowth structures than in previously studied nanoparticles. Our results introduce a class of gold bipyramid-based nanoparticles with interesting and potentially useful features to the toolbox of gold nanoparticles.

## Abstract (web summary)

Tuning the morphology of nanoparticles can alter their optical properties but often at the cost of monodispersity. Here, the authors report the synthesis of monodisperse anisotropic gold nanoparticles with various tip geometries as well as highly tunable size augmentations from purified gold bipyramids.

## Graphical abstract

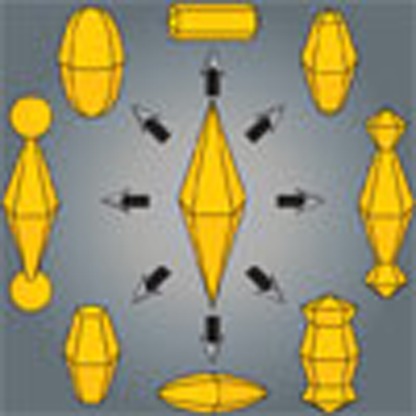

Noble metal nanoparticles have become an integral part of the emerging field of nanotechnology, with a wide variety of potential applications, including surface-enhanced Raman spectroscopy (SERS), drug delivery and therapeutics, catalysis and non-linear optics^1^,^2^,^3^,^4^,^5^. Due to the structure- and size-dependent character of localized surface plasmon resonance and local field enhancement^6^,^7^,^8^, precise control over the synthesis of nanostructures allows for a variety of programmable designs and highly tunable optical properties of the metallic metals. In addition, the ability to attain monodisperse colloidal nanoparticles in high yield is a critical step in the widespread use of these material. Gold bipyramids have shown remarkable size and shape monodispersity^9^. By theoretical calculation, stronger local field enhancement is expected in bipyramids than in nanorods or other shapes owing to the sharp tips^10^. However, the direct synthesis only yields roughly 30% bipyramids, with the other shape impurities being nanorod (∼10%) and pseudo-spherical particles (∼60%)^9^. The yield has been improved slightly using surfactant (cetyltributylammonium bromide) with larger headgroups than cetyltrimethylammonium bromide (CTAB)^11^, but pure gold bipyramids are yet unrealized using synthetic approaches alone. Recently, purification through depletion-induced flocculation has been shown to successfully separate gold nanorods from spherical nanoparticles^12^. Herein, we demonstrate purification of a range of gold bipyramid sizes through depletion flocculation using benzyldimethylhexadecylammonium chloride (BDAC) as the surfactant, and the purified product can further be used as a seed to craft other monodisperse nanoparticles (Fig. 1).

## Results

### Purifying and enlarging the bipyramids

**Figure 2:**
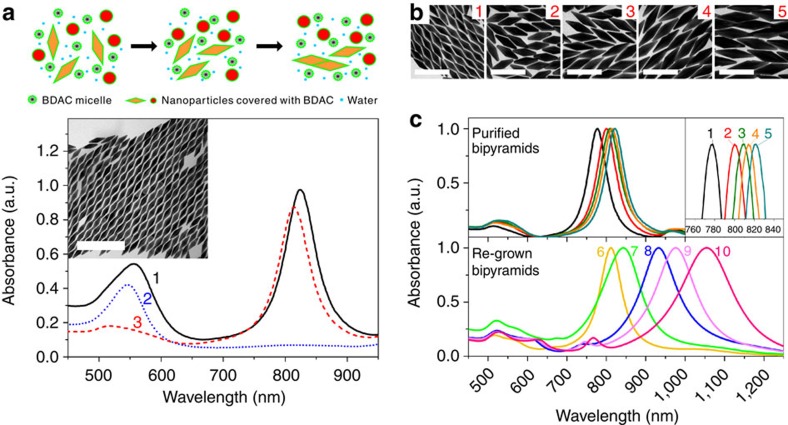
Various sizes of bipyramids generated from the purification and regrowth. (**a**) Schematic illustration, ultraviolet–vis-near-infrared spectrum and TEM image of bipyramids resultant from the purification by depletion flocculation with BDAC. (**b**) The various sizes of bipyramids are regrown from the original bipyramid seed in **a**. Bipyramids shown in **b** (1–5) correspond to spectra 6–10 in **c**. (**c**) The normalized extinction spectra of purified (1–5) and regrown bipyramids (6–10) are shown. The inset shows an enlarged spectrum of the LSPR for the purified bipyramids. Full width at half maximum of LSPR peaks were measured as 58, 60, 63, 60, 59, 73, 113, 121, 126 and 154 nm or 21.4, 20.7, 19.7, 20.7, 21.0, 17.0, 11.0, 10.3, 9.8 and 8.1 eV for 1–10, respectively.

Gold bipyramids were synthesized according to the method by Liu and Guyot-Sionnest^9^ using seed-mediated growth, and subsequently purified by depletion flocculation. BDAC was chosen for the purification due to the significantly higher micelle concentration than CTAB at the same concentrations (roughly 2.6 times more, see Supplementary Note 1). According to the theoretical model proposed by Park, the high micelle concentration induces flocculation at much lower surfactant concentrations, helping to avoid certain issues that can generally arise at high surfactant concentrations such as high solution viscosity, solubility issues and the unpredictable transitions from spherical micelles to rod-like or worm-like micelles^12^. Figure 2a shows representative ultraviolet–visible (ultraviolet–vis) spectra and transmission electron microscopy (TEM) image for highly purified gold bipyramids over 90% (see also Fig. 2c for spectra 1–5 and Supplementary Fig. 1a for other sizes). The purification utilizes depletion attraction forces to selectively flocculate nanopaticles with high facial surface area, in this case the bipyramids. The strength of the attractive force is proportional to the volume of pure water generated during the approach of the two nanoparticles, which is likewise proportional to the possible contact area of the nanoparticles. Therefore, particles with large possible contact areas, such as the bipyramids, will selectively flocculate, while those with low possible contact areas, such as the spherical impurities, will remain in the supernatant (1: as-synthesized bipyramids, 2: supernatant, 3: purified bipyramids, see also Supplementary Fig. 1 and Methods for detailed procedure and TEM images). Bipyramids over 100 nm become increasingly difficult to separate from pseudo-spherical impurities as the facial surface area of both bipyramids and pseudo-spherical impurities are increased, resulting in undesirable co-flocculation (Supplementary Fig. 1c).

On obtaining pure, monodisperse gold bipyramids, a variety of other gold nanostructures with novel shapes or changes in size were synthesized from the bipyramids in a process similar to seed-mediated growth. Because the ‘seed' used in this case is monodisperse, the product obtained through these transformations is also monodisperse in both shape and size, with the polydispersities ranging from only 2 to 5%. Using the purified bipyramid as a seed, the limitations to size of both the synthesis and separation can be overcome, allowing an increase in particle size by over 100 nm with high purity. TEM images in Fig. 2b and ultraviolet–Vis spectra 6–10 in Fig. 2c show the range of sizes of the regrown bipyramids obtained by adding either a different amount of bipyramid seeds or adjusting the concentration of reactants in growth solution (see Supplementary Tables 1 and 2 for synthetic conditions and detailed size measurements). The full width at half maximum of the longitudinal surface plasmon resonance (LSPR) peak is between 58 and 153 nm and these values, which compare favourably to that of nanorods (∼100–200 nm or 6.2–12.4 eV)^13^,^14^, also confirm the monodispersity of the bipyramids. It has also been observed that the aspect ratio can be altered slightly during the regrowth by either changing the pH or the amount of AgNO_3_ in the growth solution (Supplementary Fig. 2b).

### Bipyramid dumbbells

**Figure 3:**
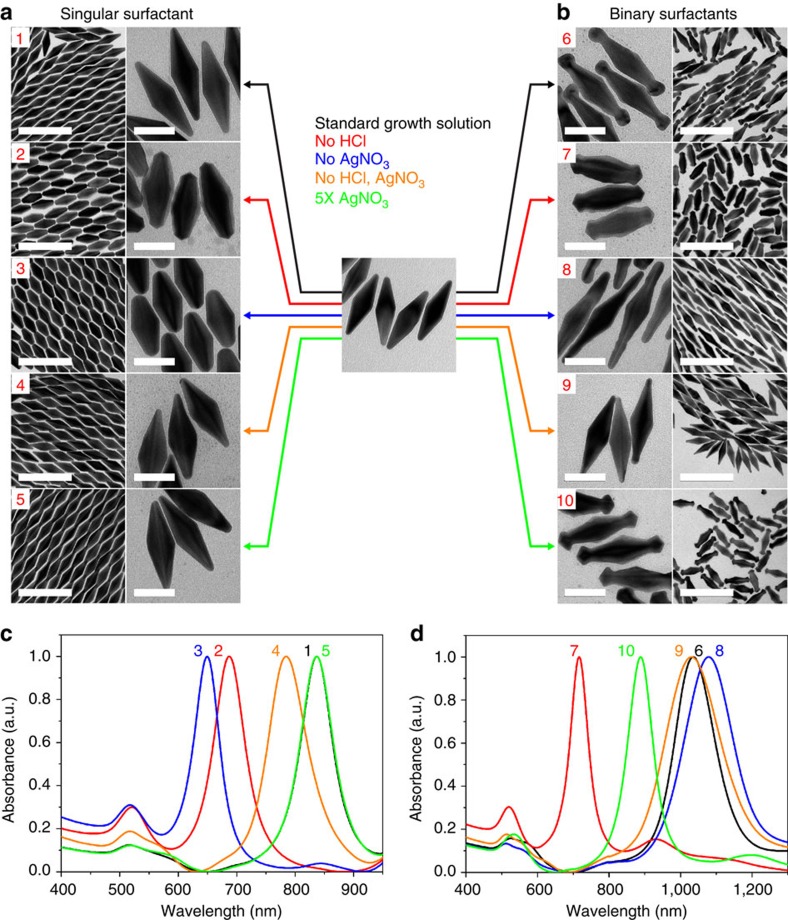
Shape-controlled bipyramids regrown with either singular and binary surfactants are shown. (**a**,**b**) TEM images of the monodisperse, regrown structures from purified bipyramid seeds with singular surfactant (conditions 1–5) and binary surfactants (conditions 6–10). The coloured arrows indicate the specific conditions for regrowth (see also Supplementary Table 1 for detailed conditions). Scale bars, 200 nm (low magnification); 50 nm (high magnification). (**c**,**d**) Normalized ultraviolet–vis–near-infrared spectra of regrown structures with singular surfactant (1–5 in **c** correspond to 1–5 in **a**) and binary surfactants (6–10 in **d** correspond to 6–10 in **b**), respectively. See Supplementary Table 3 for detailed size measurements.

In addition to enlarging the bipyramids, regrowth of bipyramids by changing the synthetic conditions introduces several new structures to be added to the toolbox of gold nanoparticles, but also offers powerful insights into the growth mechanism yet unrealized with nanostructures of simpler shape. Regrowth of gold nanospheres and gold nanorods is well studied, and the transformation of nanorods to dumbbells has revealed much about the intricacies of nanoparticle growth conditions. However, both the nanosphere and nanorod fail to offer the complex crystal facets and twinned shape that the bipyramid provides. We first compared the regrowth of bipyramids using different surfactants (CTAB, cetyltrimethylammonium chloride (CTAC) and BDAC) as well as their mixtures. For standard growth solution with individual surfactants, size augmentation was dominant over any structural changes. Size augmentation of bipyramids with CTAB provided the best shape uniformity and surface smoothness, compared with both BDAC and CTAC (Fig. 3a, condition 1, and Supplementary Fig. 3). These results can be explained by considering the different binding affinities of CTAB and BDAC, that is, Br^−^>Cl^−^, and also the degree of underpotential deposition in the presence of Ag^+^ ions onto the gold surface^9^,^15^,^16^,^17^. The regrowth with CTAC resulted in random shape growth and aggregation because centrifugation with CTAC to remove remaining CTAB reduces particle stability, which is consistent with reported result for nanorod growth using CTAC (Supplementary Fig. 3)^18^,^19^. According to reports of nanorod growth using cationic headgroup with bromide anion as a counterpart, larger headgroups lead to nanorods of higher aspect ratio and slowed growth rate, implying a higher binding affinity and resulting in a more stable bilayer on the particle surface^11^,^15^,^20^,^21^. The results from our observation and literature suggest that the affinity of surfactants in the presence of Ag^+^ ions could be extended to CTAB>BDAC>CTAC.

Systematic studies were conducted with the three possible binary surfactant combinations, that is, CTAB/CTAC, CTAB/BDAC and BDAC/CTAC (Supplementary Fig. 4). Despite changing the molar ratios, overgrowth at the tips was not observed and only somewhat observed for BDAC/CTAB or CTAC/BDAC, respectively (Supplementary Fig. 4a). Intriguingly, the molar ratio of surfactants in binary systems showed a huge influence on the regrowth of bipyramids, especially at the tip region. Previously, mixed surfactants as capping agents have been used for adjusting the aspect ratio of gold nanorods^15^,^21^, Ag-tipped overgrowth of gold nanorods^22^ and tetrahedral-like gold nanotripods^23^. However, the role of each component for the growth is still ambiguous due to the complexity of interactions. As mentioned previously, the binding affinity of CTAB is greater than that of CTAC. However, as the ratio of CTAC:CTAB is increased to between 90:1 and 900:1, an equilibrium exists where CTAC occupies some of the surface area. Evidently, the CTAC is localized to the tip region where the binding preference of CTAB over CTAC is minimal. In this case, growth at the less protected ends capped by CTAC results in the observed tip overgrowth utilizing the shape-directing properties of the halide (Fig. 3b, condition 6)^15^,^16^,^17^,^22^. When the ratio is either too high or too low or when pure CTAB is used, the tip growth is not observed as size augmentation becomes the dominant growth mode as the surface is covered uniformly in a single surfactant (Fig. 3a, condition 1, and Supplementary Fig. 4b,c). This experimental observation is qualitatively consistent with reported results for adjusting aspect ratio of gold nanorod and Ag-tipped overgrowth of gold nanorods using a BDAC/CTAB mixed surfactant system^15^,^22^.

By carefully modifying the reaction conditions, we were also able to control the tip shape of dumbbell-like bipyramids to further confirm the role of components in the growth solution. Figure 3 shows TEM images and ultraviolet–vis–near-infrared spectra of a variety of monodisperse regrown structures from bipyramids with singular or binary surfactants. We observed marked changes at the bipyramid tips including spherical-, rod- and bipyramid-like tips with binary surfactants. The standard growth solution of bipyramids in these experiments consisted of 91.9 μM of HAuCl_4_, 18.4 μM of AgNO_3_, 0.0184 N of HCl and 147 μM of L-ascorbic acid. The molar ratio of [ascorbic acid]/[HAuCl_4_] was 1.6 in Fig. 3 to ensure complete reduction of Au^3+^ to Au^0^ (ref. 22). If less ascorbic acid is used, remaining Au^3+^ acts as an oxidant, and an etching process becomes dominant, resulting in shortened bipyramids (Supplementary Fig. 5)^24^. The major factors for the shape changes in these reactions were HCl and AgNO_3_. It is reported that the reduction rate of Au^3+^ can be increased by increasing the pH of the solution, which can cause fast deposition along the existing crystal face, leading to growth of the nanorod in all directions having shorter aspect ratio^25^,^26^. The pH of the growth solution is 1.74 for the case of standard growth solution and 3.62 for the growth solution without HCl. Under both of these conditions, reduction of Ag^+^ can be neglected due to the increased redox potential of ascorbic acid^27^. It is now established that a monolayer of silver can be deposited onto a particular surface of gold during the growth through underpotential deposition, thus stabilizing the surface and slowing the growth rate^9^,^16^,^17^,^28^. The growth rate of all facets in the structure can be altered depending on the degree of surface coverage by underpotential deposition of silver ions, and therefore differing growth rates of particular facets will determine the final structure of the nanoparticle.

The results in Fig. 3a, condition 2, and Fig. 3b, condition 7, show predominant growth along the short axis with sharp apices. As compared with growth in standard growth solution, a faster growth rate is expected in all directions for bipyramids without HCl at pH 3.62. However, the growth rate at the tip is greatly inhibited, because of the presence of Ag^+^ ions, which can more easily access the tip region, resulting in underpotential deposition. The high curvature of the tip region results in lower surfactant packing density than on the bipyramid faces, allowing additional room for Ag^+^ to deposit in the intersurfactant regions^22^. This is resulted in a stepped facet formed along the long axis of the bipyramids, thereby sharpening the bipyramid tip for both^9^. The growth for binary surfactants showed increased overgrowth at the tip than for singular case because it has less coverage of CTAB layer accelerating the growth along the tip region. For binary surfactants, adding more or less AgNO_3_ without HCl resulted in little enlogation and unrestricted growth of the width, especially at the tips, indicating that increased inhibition of growth along the long axis can greatly alter the shape of particle (Supplementary Fig. 6a)

Likewise, in the experiments without AgNO_3_ at pH 1.76, fast growth is expected due to the absence of Ag^+^ ions, however, slower than the case without both HCl and AgNO_3_ due to the effect of the pH on the reducing reduction power of ascorbic acid. The reaction proceeds with a rate that allows for controlled growth due to the low pH, but the absence of Ag^+^ ions, normally yielding a stepped crystal facet at this pH for bipyramids, results in facilitated growth of a nanorod-like structure^9^,^16^,^17^. Without Ag^+^ ions to block certain growth facets, bipyramids grown in a singular CTAB surfactant system were synthesized with low aspect ratio (∼2.3) and poorly defined tips; due to the even coverage of the singular surfactant and lack of Ag^+^ underpotential deposition, growth occurred evenly in all directions (Fig. 3a, condition 3). Interestingly, rod-like tips were formed in the binary surfactant system (Fig. 3b, condition 8). Similar to the above case, it is believed that overgrowth at the tip region was induced from the reduced surface coverage of CTAB in the binary system. In the absence of Ag^+^, the overgrowth was accelerated and bypassed the stepped structure to form the rod-like structures at the tips.

Figure 3a, condition 4, and Fig. 3b, condition 9, show the regrowth of bipyramids without both HCl and AgNO_3_. In this case, a much faster growth rate is expected than the former two cases, either without HCl or without AgNO_3_. Due to a greatly faster deposition and the absence of Ag^+^ inhibition, rapid addition of Au^0^ atoms occurs on the stepped surface of bipyramids evenly in all directions. This results in particle maintaining its original bipyramid shape for both the singular and binary surfactant systems. The aspect ratio for binary surfactants was slightly higher than the singular case because of the lessened CTAB coverage as mentioned above, resulting in a small preference for growth at the tip and a slightly elongated particle.

Meanwhile, additional AgNO_3_ (5 × more than standard growth conditions) shows significant morphological changes at the tip region in binary surfactants, given less coverage of CTAB at the tip than the singular surfactant. Distinct crystalline structures identical to bipyramids were formed at the tips at both ends (Fig. 3b, condition 10). However, negligible changes were observed from singular CTAB surfactant (Fig. 3a, condition 5). These results indicate that insufficient protection from CTAC surfactant can be more sensitive for inhibition of growth from underpotential deposition of Ag^+^, allowing for easier access to the tip end, resulting in significant shape changes. The addition of more acid in this case results in negligible changes for both singular and binary surfactants (Supplementary Figs 2 and 6b). The ultraviolet–vis–near-infrared spectra in Fig. 3c,d reflects the various structural changes of regrown bipyramids dependent on the growth conditions. Because most of regrown structures show changes in length, significant peak shift of longitudinal plasmon peaks with narrow widths were observed.

**Figure 4:**
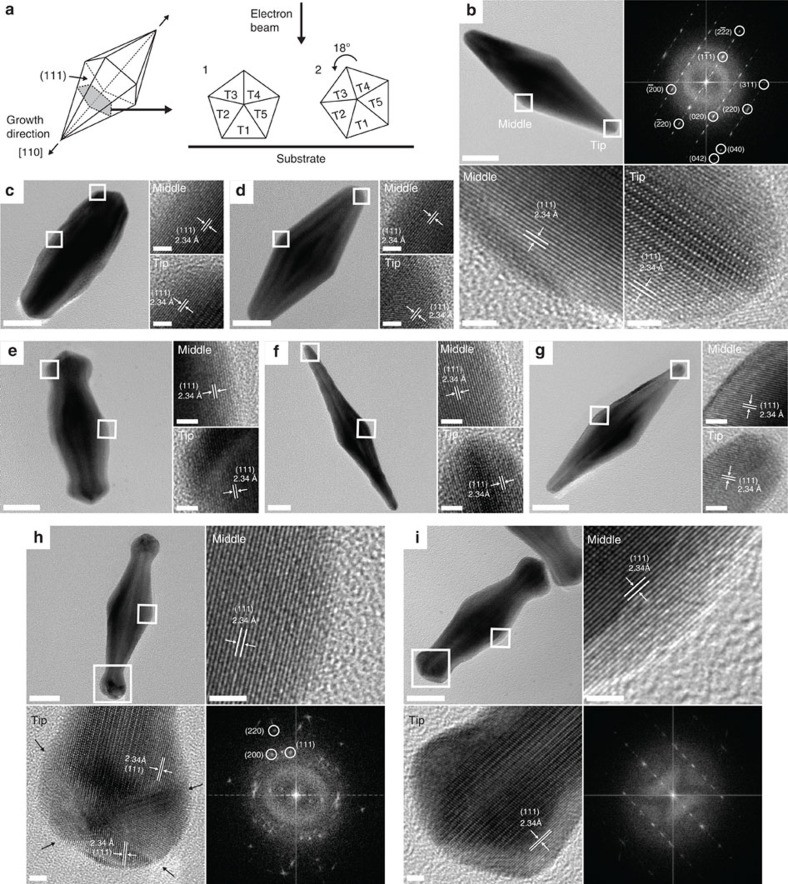
HR-TEM images and FFT patterns of regrown bipyramids with singular and binary surfactants. (**a**) Schematic illustrations for cyclic penta-tetrahedral twinning of bipyramids. The grey area shows the cross-section of the bipyramid perpendicular to the growth direction. Each twinning plane is labelled from T1 to T5. Schematic on the right of the panel shows most of the possible orientations of bipyramids on the substrate with respect to the beam direction. Regrown bipyramids in Fig. 3a—conditions 1–3 for **b–d**, respectively; Fig. 3b—conditions 7–9 for **e**–**g**, respectively; Fig. 3b—conditions 6 and 10 for **h** and **i**, respectively. Magnified images showing the lattice fringes represent the areas marked with white boxes at the tip and middle of particles. The black arrows in **h** indicate the twin planes of the spherical tips in the dumbbell-shaped structures. Scale bars, 20 nm (low-magnification images); 2 nm (high-magnification images).

The crystalline structure of individual regrown bipyramids with singular and binary surfactants was determined by high-resolution TEM (HR-TEM) and fast Fourier transform (FFT) patterns (Fig. 4). Figure 4b shows HR-TEM images and the corresponding FFT patterns of enlarged bipyramids resulting from the regrowth with a singular surfactant and standard growth solution. The magnified images in Fig. 4b show clear lattice fringes at the tips and middle of the particles. It shows that both fringes are parallel to the growth axis and twinned along the long axis. The spacing between lattice fringes is confirmed as 0.234 nm by direct measurement, corresponding to the (111) facets of the bipyramids^9^. The FFT pattern in Fig. 4b shows a diffraction pattern of the bipyramid corresponding to orientation 1 in Fig. 4a. The indexed reflections in the FFT pattern correspond to the lattice parameters: *d*_111_=0.234 nm, *d*_220_=0.143 nm, *d*_222_=0.118 nm, *d*_200_ and *d*_020_=0.204 nm, *d*_311_=0.122 nm, *d*_400_=0.102 nm, and *d*_420_=0.091 nm. Reflections indexed as 
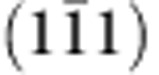
, (311) and (220) are scattered from T3 and T4. Reflections indexed as 
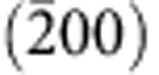
, (020) and (220) are from T1. The remaining reflections that are not indexed are induced by multiple scattering effect. All measured values are within an error range of ±2% compared with bulk data and agree well with reported results, indicating that regrown bipyramids are penta-twinned with face-centred cubic structure^29^,^30^,^31^,^32^. All of the regrown bipyramids with either singular or binary surfactants are confirmed to have the same FFT pattern as a bipyramid in Fig. 4b (Supplementary Fig. 7). The tip angles (*θ*) of bipyramids in Fig. 4b were measured to be 27.02±1.35°, which correspond to (117) high-index facets having an average step length (*s*) of ∼3.5 atoms (Supplementary Fig. 7a)^9^. However, the identity of this high-index facet varies as the tip angles differ from that of bipyramids^9^,^33^,^34^,^35^. If the tip angle is smaller than bipyramids in Fig. 4b, the average step length (*s*) can be >3.5, which can be indexed as {11*l*}, where *l*>7. Likewise, when the tip angle is larger than that of bipyramids, the average step length (*s*) can be <3.5, which can be indexed as {11*l*}, where *l*<7. Interestingly, HR-TEM images and FFT of the dumbbell-like bipyramid in Fig. 4h show multiply twinned spherical structures at both tips. (The black arrows in Fig. 4h indicate the twin planes of the spherical tips.) The circular reflections in Fig. 4h correspond to the lattice parameters: *d*_111_=0.229 nm, *d*_220_=0.143 nm and *d*_200_=0.203 nm, respectively. This circular type of FFT pattern resembles spherical particles having pentagonal symmetry, also known as decahedra^29^,^30^,^32^,^36^.

### Oxidative etching of bipyramids

**Figure 5:**
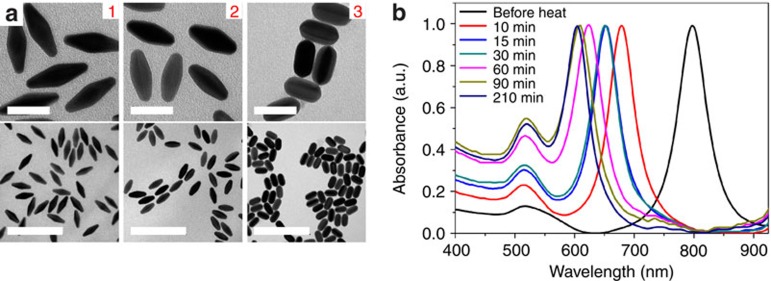
Etched structures from oxidative etching with BDAC surfactant and heating at 120 °C. (**a**) TEM images of the etched structures resulting from heating for 10, 30 and 90 min, shown from 1 to 3. Scale bars, 50 nm (high magnification); 200 nm (low magnification). (**b**) Normalized ultraviolet–vis–near-infrared spectra of etched structures resultant of increasing the heating time.

Gold bipyramids can also undergo oxidative etching utilizing molecular oxygen dissolved in solution. Cycling oxidations and reductions of gold atoms can slowly shape the nanoparticle through a time-dependent aging process^37^. Additive oxidants such as hydrogen peroxide have been shown to accelerate the etching process and to reshape nanoparticles through atomic substraction and addition, but result in nanoparticles with poor shape and size dispersity^11^,^38^. Taking advantage of the purity of the gold bipyramids and their inherent monodispersity, this etching process has been adapted to create other monodisperse structures. Because of the high-energy nature of the bipyramid facets and sharp tips, these highly reactive particles are susceptible to oxygen as an etchant despite it being a weak oxidant. As seen in Fig. 5a, bipyramids begin to etch at the tips and are continuously sculpted to rice shaped and eventually rod shaped as the reaction progresses at 120 °C and in the presence of 0.1 M BDAC. Ultraviolet–vis spectra in Fig. 5b show continuous changes that are consistent with the structural changes as seen in the TEM images. In the presence of BDAC as a protective agent and etchant, the purified gold bipyramids facilitate a four-electron oxidation of four Au^0^ atoms to Au^+^, forming the gold chloride salt and simultaneously reducing molecular oxygen to water. The high temperature of the system then allows for disproportionation at the surface of the nanoparticle, depositing Au^0^ atoms back onto the surface in low-energy and low-defect areas^37^. This process cycles, continuously removing atoms at defect areas and replacing them in defect-free areas, until the particle reaches an energy minimum, in this case a gold nanorod. At room temperature, a similar kinetic process still occurs, but resulting in a blue-shift of the LSPR peak of less than a couple nanometres every day (Supplementary Fig. 8a). This indicates the thermodynamic nature of the reaction, and that the etching of the bipyramid to the nanorod could be further accelerated by increasing the temperature. When CTAB is used as a protective agent and etchant, the reaction slows, requiring nearly an order of magnitude longer to obtain the analogous structures to the ones obtained from heating for 30 mins in Fig. 5a (Supplementary Fig. 8b). These reaction rates are qualitatively consistent with generally accepted reactivates of halides^37^. It is noteworthy that the etching reaction rate can be increased by decreasing the concentration to the critical micelle concentration of the surfactant (1 mM), which results in lessened protection of the nanoparticles from the oxidative species (Supplementary Fig. 8c).

**Figure 6:**
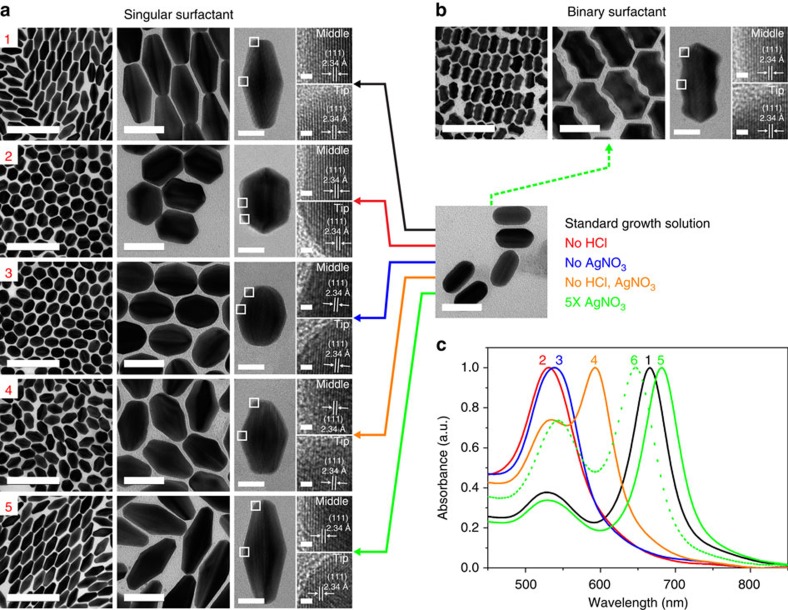
Controlling the shape of oxidatively etched nanorods through the regrowth with singular and binary surfactants. (**a**,**b**) TEM and HR-TEM images of monodisperse, regrown structures from the oxidatively etched nanorods in Fig. 5a (90 min heating) with singular surfactant (**a**) and binary surfactants (**b**). The coloured arrows indicate the specific conditions for regrowth (See also Supplementary Table 1 for detailed conditions). Magnified images showing the lattice fringes represent the areas marked with white boxes at the tip and middle of particles. (**c**) Normalized ultraviolet–vis–near-infrared spectra of the regrown structures with singular surfactant (1–5 correspond to 1–5 in panel a) and binary surfactants (6 corresponds to **b**) are shown. See Supplementary Table 3 for detailed size measurements. Scale bars, 200, 50, 20 and 1 nm (from low to high magnification respectively).

To extend our regrowth strategy to other structures, we further synthesize regrown structures using the monodisperse etched structures as seeds with both singular and binary surfactants. Figure 6 shows TEM images and ultraviolet–vis spectra of regrown structures using the rod shape particles from etching as seeds. The growth behaviours from etched particles using unitary and binary surfactants were confirmed to be very similar, likely to due to the absence of the sharp tips that affect the tip of gold bipyramids. Interestingly, the rice shape particles from the etching can be reversed to the original structure of bipyramids when the standard growth solution is used for the regrowth (Supplementary Fig. 9). On the other hand, the regrowth of rod shape particles with standard growth solution cannot be fully reversed to the bipyramids, instead forming a bifrustum, ultimately lacking the sharp tips of a bipyramid (Fig. 6a condition 1). Applying the same growth conditions as bipyramids, new types of nanostructures from bifrustum to short dumbbell were synthesized using the etched particles, with plasmonic resonances covering the short wavelength region between 500 and 800 nm. All of the regrown structures from the etched rod-shaped particles using singular or binary surfactants are also confirmed to have similar FFT patterns as the bipyramids in Fig. 4b (Supplementary Fig. 7).

## Discussion

The regrowth strategy we have described has successfully synthesized various, novel gold nanoparticle geometries based on the bipyramid shape. The purity and monodispersity of the bipyramid results in a likewise pure and uniform product ranging from augmented bipyramids, dumbbells with spherical, pointed, and rod-like tips, bifrustums and spherical polyhedra. Regrowth of bipyramids in a single surfactant can be controllably tuned to give a length of well over 200 nm and a longitudinal plasmonic resonance peak well into the near-infrared range, as well as allowing for control of the aspect ratio. Regrowth with binary surfactant systems offer insight into the localization of particular surfactants on the particle surface. When combined with varying concentrations of acid and silver offer the opportunity to yield unique geometries. In addition, the high-energy nature of the bipyramid structure allows for controllable oxidative etching using only molecular oxygen as the etchant to create highly monodisperse nanorice and nanorod structures, which have additionally been regrown using the same procedure. Finally, the various growth conditions starting from the highly monodisperse but twinned structure of the bipyramids allows to create noble metal nanoparticles of a wide range of size and shape with unprecedented narrow distribution, and this will be key in future efforts to assemble these structures for shaping the optical response for potential applications in self-assembly, non-linear optics and surface-enhanced Raman spectroscopy (SERS).

## Methods

### Materials and instruments

All chemicals were purchased from commercial suppliers and used without further purification. CTAB (Bioxtra, ≥99.0%), BDAC (cationic detergent), cetyltrimethylammonium chloride, citric acid trisodium salt dihydrate (≥99.5%, BioUltra, for molecular biology) (CTAC, ≥98.0%), hydrogen tetrachloroaurate trihydrate (HAuCl_4_·3H_2_O) and L-ascorbic Acid (Bioxtra, ≥98.0%) were purchased from Sigma Aldrich. Silver nitrate (AgNO_3_, ≥99.8%) and sodium borohydride (NaBH_4_, ≥99%) were purchased from Fluka. Hydrochloric acid (HCl, 1 N) was purchased from Fisher scientific. Nanopure water (18.2 MΩ, Barnstead Nanopure, Thermo Scientific, MA, USA) was used in all experiments. The glass vials were purchased from Kimble chase (4 and 20 ml, NJ, USA). All glasswares were cleaned using freshly prepared aqua regia (HCl:HNO_3_ in a 3:1 ratio by volume) followed by rinsing with copious amounts of water. RCT Basic (IKA, NC, US) was used for magnetic stirring. Ultraviolet–vis–near-infrared spectra were measured with Synergy H4 (Biotek, VT, USA) and Cary 5000 ultraviolet–vis–near-infrared (Agilent, CA, USA). The formvar/carbon-coated copper grid (Ted Pella, Inc. Redding, CA, USA) and TEM (Tecnai G2 F30 Super Twin microscope, 300 kV and Tecnai G2 Spirit, 200 kV, FEI, OR, USA) were used for the TEM analysis.

### Synthesis and purification of gold bipyramids

The gold bipyramids were synthesized according to the literature procedure^9^. Briefly, gold seeds were prepared with 18.95 ml of ultrapure water, 0.25 ml of 10 mM HAuCl_4_ and 0.5 ml of freshly prepared 10 mM sodium citrate, followed by adding 0.3 ml of fresh and ice-cold 10 mM NaBH_4_ under 500 rpm with magnetic stirrer at room temperature. The reaction mixture was stirred for 2 h, and aged for a week before use (After aging, seed solutions are stable for a month and give reproducible results for the synthesis of bipyramids). The bipyramids were grown in a solution containing 10 ml of 0.1 M CTAB, 0.5 ml of 0.01 M HAuCl_4_, 0.1 ml of 0.01 M AgNO_3_, 0.2 ml of 1 N HCl, 0.08 ml of 0.1 M ascorbic acid, and varying amounts of seed solution; shown were seed volumes of 110, 100, 95, 80 and 60 μl corresponding to 1–5 in Supplementary Fig. 1a and Supplementary Table 2. The solution was gently stirred at 400 r.p.m., and was kept in an oil bath at 30 °C for 2 h. The colloid was centrifuged at 13,000 g at 30 °C for 15 min, and washed with 10 ml of 1 mM CTAB, performed twice. After removing the supernatant, the precipitate was redispersed in 3 ml of 1 mM CTAB solution for further purification. Volumes of 0.5 M BDAC solution and ultrapure water were added to the 3 ml of crude bipyramid solution to obtain 10 ml of solution with the desired BDAC concentration. The concentration of BDAC desired is dependent on the size of the gold bipyramids and was determined experimentally (Supplementary Fig. 1). Concentrations of BDAC used were 230, 260, 310, 320 and 350 mM corresponding to the bipyramids prepared with 60, 70, 95, 100 and 110 μl of seed solutions, respectively. The solution was mixed and left undisturbed in an incubator at 30 °C for 11 h. The resulting pink supernatant was carefully removed, and 3 ml of 1 mM CTAB was added to the vial to redisperse the precipitate. The vial was then sonicated for 1 min. The resulting purified solution (brown in colour) was centrifuged at 8,000*g* for 8 min and washed with 1 ml of 1 mM CTAB, repeated twice, to remove the excess BDAC. Finally, the purified bipyramids were redispersed in 1.5 ml of 1 mM CTAB solution to be used for all regrowth reactions. The purified bipyramids in Fig. 2a prepared with 80 μl of seed solution were used as seeds for all regrowth reactions.

### Controlling the size of bipyramids

The size of gold bipyramids can be controlled using either a different concentration of growth solution or different amount of purified seed solution. For the typical preparation of regrowth solution, 0.9 ml of 0.1 M CTAB solution was kept for 5 min in an oil bath at 30 °C with magnetic stirring at 400 r.p.m. HAuCl_4_, AgNO_3_, HCl and ascorbic acid were then added sequentially as detailed in Supplementary Table 1 and kept for 5 min. Finally, an amount of purified bipyramid seed solutions (Supplementary Table 1) was added and kept for 2 h. To maintain the reaction volume constant, the varied amount of purified bipyramid seed solutions were adjusted to 0.1 ml with 1 mM CTAB. The resulting solution was centrifuged at 7,000*g* for 8 min and washed with 1 mM CTAB, repeated twice, then redispersed in 1 mM CTAB for further characterization.

### Controlling the shape of nanoparticles

Various tips and unique shape of structures can be controlled with single or binary surfactants along with modified growth solutions as detailed in Supplementary Table 1. The procedure with a single surfactant is similar to the enlarging of the bipyramid, except the condition adding chemicals. For the synthesis without HCl, AgNO_3_ or both, the same volume of nanopure water is added to the regrowth solution in its stead. For excess amount of AgNO_3_, a higher concentration of solution is used with the same volume as the standard regrowth solution. Hundred microlitre of purified bipyramid seed solution in 1 mM CTAB is added to the prepared regrowth solution and kept for 2 h. For the typical preparation of regrowth solution with binary surfactants, 0.1 M CTAC was kept for 5 min in an oil bath at 30 °C with magnetic stirring at 400 r.p.m. Hundred microlitre of purified bipyramid seed solution is centrifuged at 7,000*g* and redispersed in 0.1 ml of desired concentration of CTAB solution to adjust the ratio between the surfactants (CTAC and CTAB). The reactants with purified bipyramid seed were added same as above and kept at 30 °C for 2 h to complete the reaction. The resulting solution was centrifuged at 7,000*g* for 8 min and washed with 1 mM CTAB, repeated twice, then redispersed in 1 mM CTAB for further characterization.

### Oxidative etching of bipyramids

For oxidative etching, 100 μl of purified bipyramids in 1 mM CTAB was added to 900 μl of 100 mM BDAC solution with no other reagents. A glass vial with screw cap was sealed with teflon tape to prevent the leakage of vapour during the heating. The sealed vial was placed in oil bath pre-heated at 120 °C and kept under stirring at 300 r.p.m. with magnetic stirrer. (Use caution when heating the sealed container as pressure will build inside the flask. Properly sealing the vial is also crucial to controlling the reaction speed, as any leaks will accelerate the reaction.) After 10, 15, 30, 60, 90 and 210 min, the vials were cooled to room temperature with a water bath to halt the oxidative process. The resulting solution was centrifuged at 8,000*g* for 8 min and washed with 1 mM CTAB, repeated twice and then redispersed in 100 μl of 1 mM CTAB for further regrowth and characterization. See Supplementary Table 1 for the detailed conditions for regrowth.

## Additional information

**How to cite this article:** Lee, J.-H. *et al.* Bipyramid-templated synthesis of monodisperse anisotropic gold nanocrystals. *Nat. Commun.* 6:7571 doi: 10.1038/ncomms8571 (2015).

## Supplementary Material

Supplementary Information Supplementary Figures 1-9, Supplementary Tables 1-3, Supplementary Note 1 and Supplementary References.

## Figures and Tables

**Figure 1:**
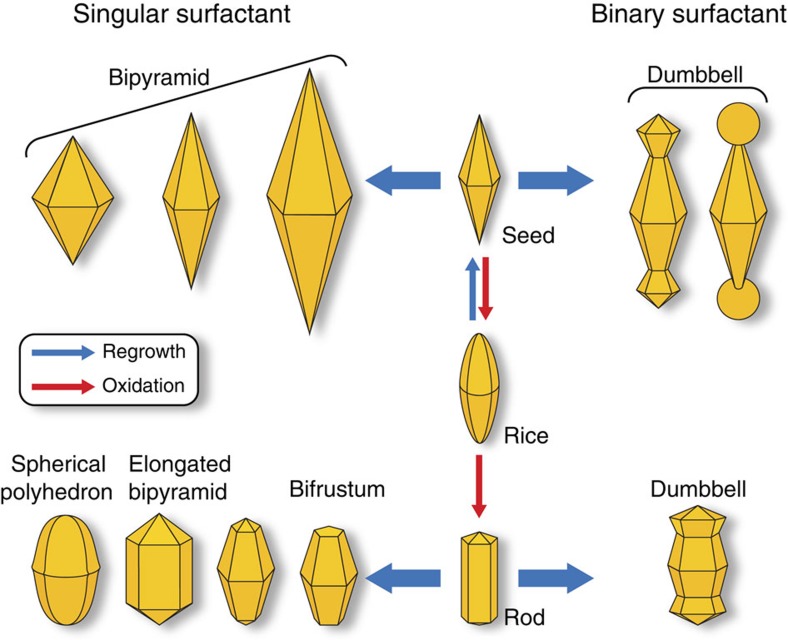
Schematic representation of the shape control of the bipyramids. The various pathways to generate unique structures originating from the bipyramid are shown.
