# Supplementary material for: Bipyramid-templated synthesis of monodisperse anisotropic gold nanocrystals
Source: Nat Commun. 2015 Jun 26;6:7571. doi: 10.1038/ncomms8571 (PMC4491807; doi:10.1038/ncomms8571)
Supplement: Supplementary Information — Supplementary Figures 1-9, Supplementary Tables 1-3, Supplementary Note 1 and Supplementary References. [file ncomms8571-s1.pdf]

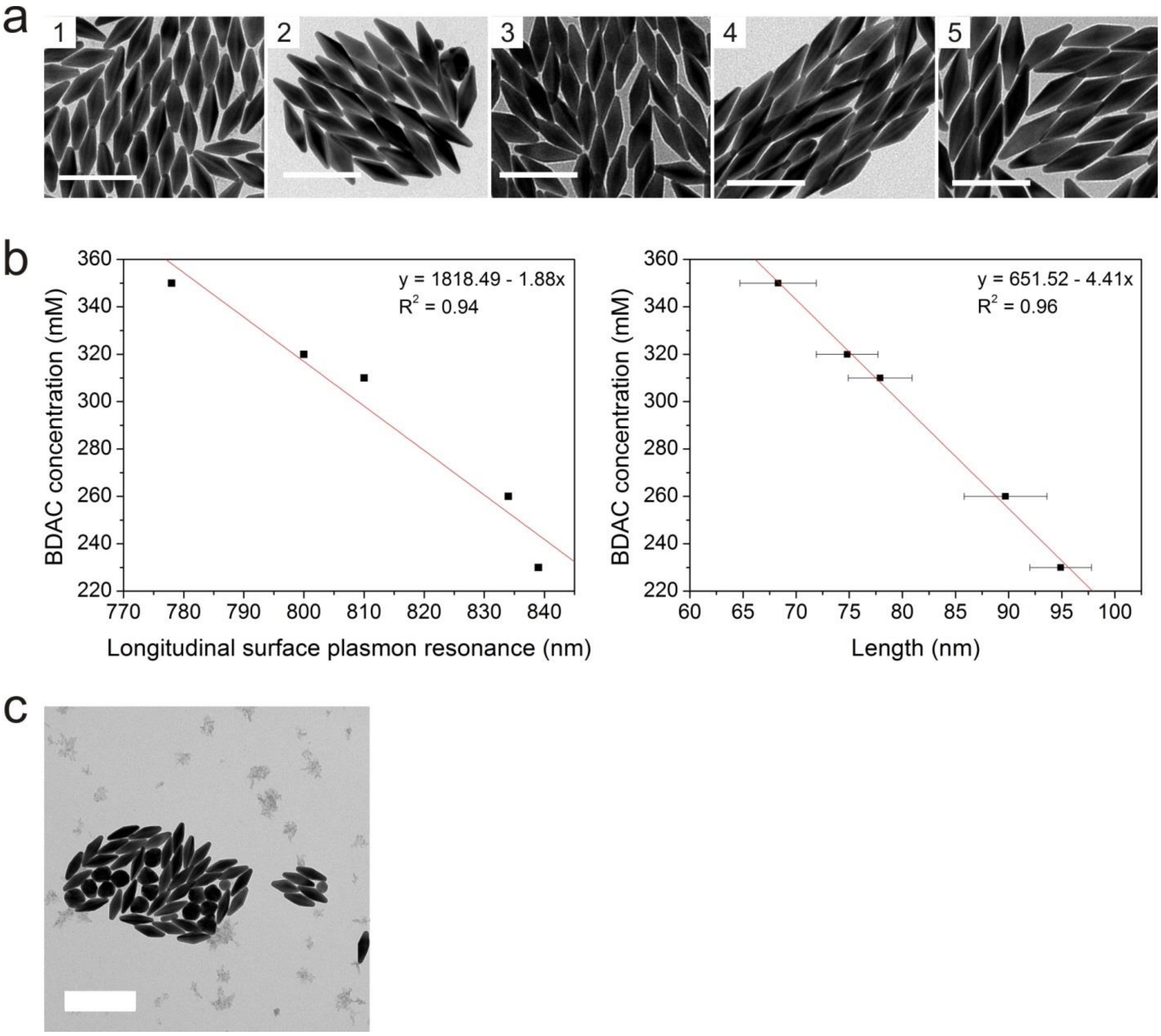

Supplementary Figure 1. **Purification of bipyramids. a**, TEM images of purified bipyramids (1-5 corresponding to 110, 100, 95, 70 and 60 µL of seed solutions). Scale bars are 100 nm. **b**, BDAC concentrations necessary for purification as a function of longitudinal surface plasmon resonance (LSPR) and length of bipyramid are shown. **c**, Co-flocculation of both bipyramids and pseudo-spherical impurities when the purification is attempted with bipyramids synthesized >100 nm. Scale bar is 200 nm. See also Supplementary Note 1. for detailed explanations.

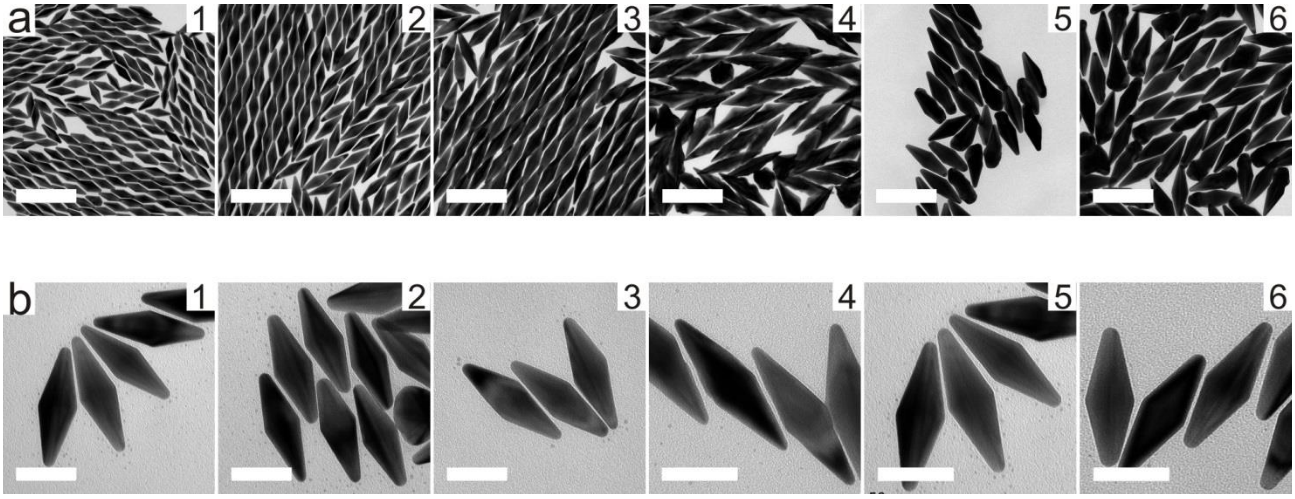

Supplementary Figure 2. **Regrown bipyramids with varying H^+, Ag^+ and seed concentrations. a**, TEM images of enlarged bipyramids resulting from regrowth with different concentration of reactants using 100 µL seeds (1-4) and 5 µL seeds (5-6). **b**, TEM images of regrown-bipyramids with different amount of $AgNO_3$ (1-3) and HCl (4-6). See also Supplementary Table 1 for detailed synthetic conditions. It is worth noting that a high concentration of gold precursor (>10 mM) in the growth solution containing 0.1M CTAB can cause the formation of CTAB-Au complex (orange color) that can affect the crystalline structure of the bypiramids, resulting in a rough surface (1-4 in panel a). Additionally, a volume of seed solution too small can cause erratic growth and yield undesired shape impurities (5-6 in panel a). All scale bars in a and b are 200 nm and 50 nm, respectively.

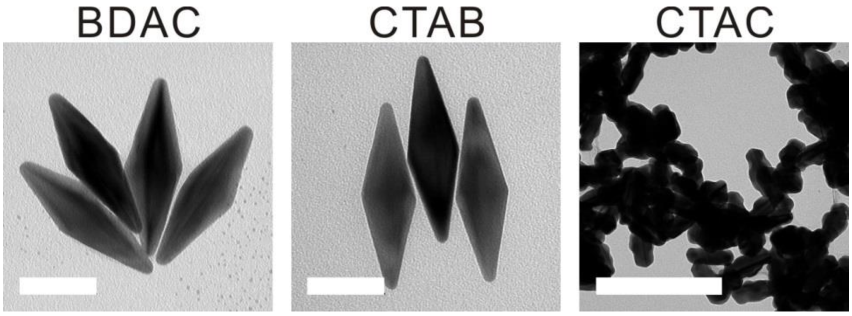

Supplementary Figure 3. **Comparison of regrown structures when varying the singular surfactant.** The growth solution consists of 4 mM $HAuCl_4$, 4 mM $AgNO_3$, 1 N HCl, and 40 mM ascorbic acid in the presence of 0.9 mL of 0.1 M CTAB, BDAC or CTAC. For the preperation of seeds for regrowth in BDAC or CTAC, 100 µL of bipyramid seed was centrifuged at 8,000 g for 8 min and washed with 1 mM of BDAC or CTAC, repeated twice, then redispered in 100 µL of 1 mM of BDAC or CTAC for further regrowth. The scale bar in the BDAC and CTAB images is 50 nm, and 200 nm for the CTAC image.

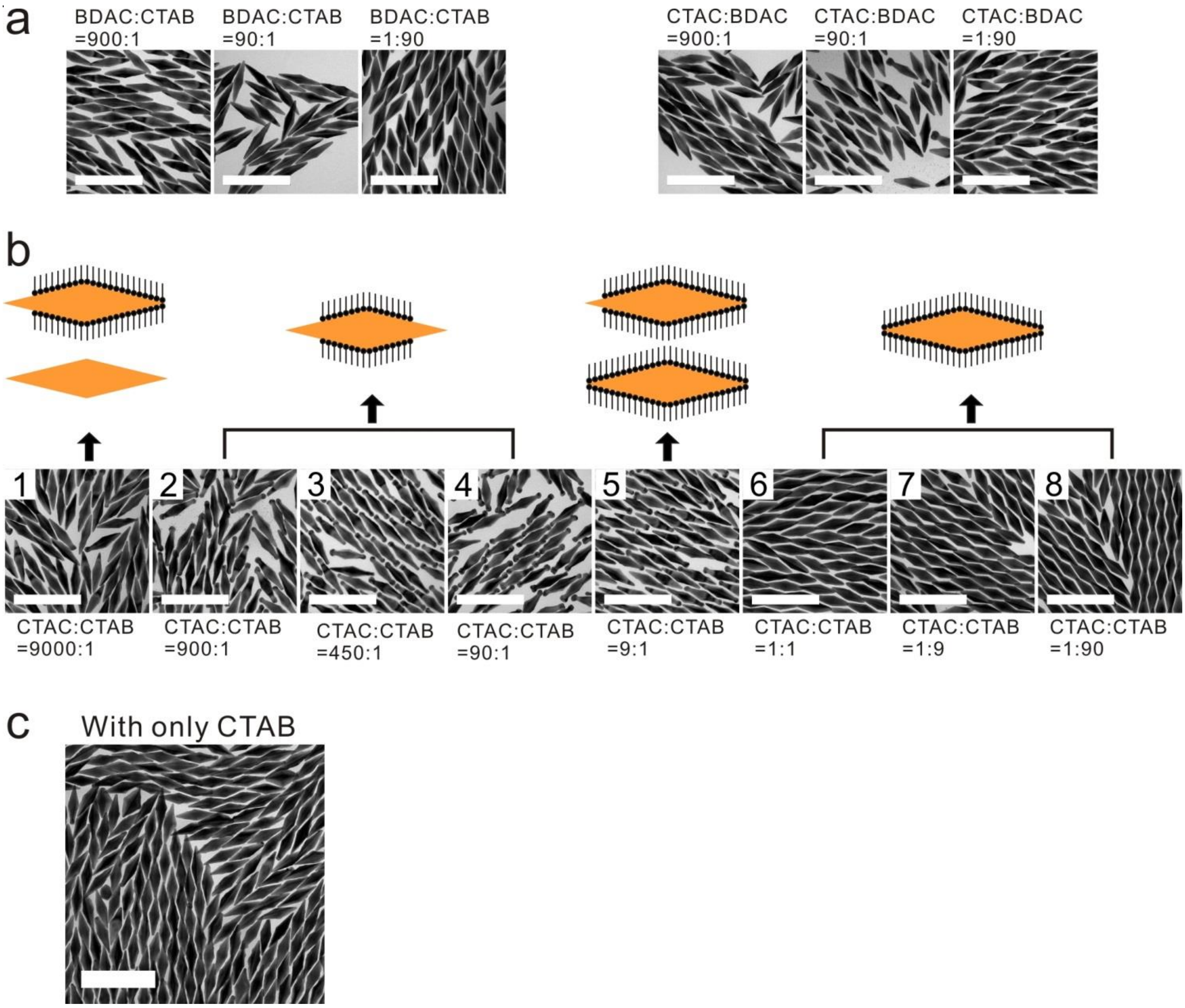

Supplementary Figure 4. **Systematic studies of the three possible binary surfactant combinations and ratios.** The growth solution consists of 2 mM $HAuCl_4$, 2 mM $AgNO_3$, 1 N HCl and 20 mM ascorbic acid for all syntheses shown in this figure. **a**, TEM images of the regrown bipyramids with BDAC/CTAB and CTAC/BDAC binary surfactant conditions. **b**, TEM images of the regrown bipyramids with CTAC/CTAB binary surfactant condition and illustrative schematic propsing the binding of the surfactant to the bipyramid surface. The black surfactant represents the CTAB while the exposed area is representative of the surface area binded by CTAC, which is likely responsible for the specific growth at the tips due to its weaker binding. **c**, TEM image of the regrown bipyramids using an identical amount of CTAB in b-4 without CTAC or BDAC, only a minimal amount of tip overgrowth was observed. All scale bars are 200 nm.

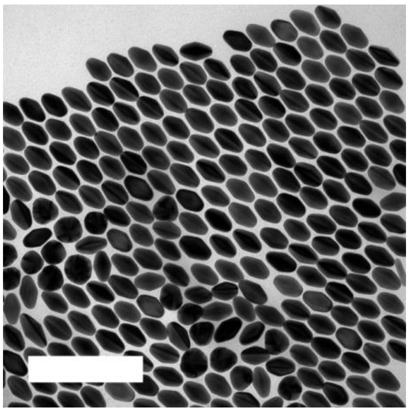

Supplementary Figure 5. **TEM image of oxidatively etched bipyramids using Au^{3+} remaining from the growth solution.** The regrowth solution consists of 2 mM $HAuCl_4$ (50 µL), 2 mM $AgNO_3$ (10 µL), 1 N HCl (20 µL) and 10 mM ascorbic acid (8 µL) with 0.9 mL of 0.1 M CTAB. Note that the molar ratio of [ascorbic acid]/[$HAuCl_4$] in this case is 0.8 compared with 1.6 in standard growth solution.

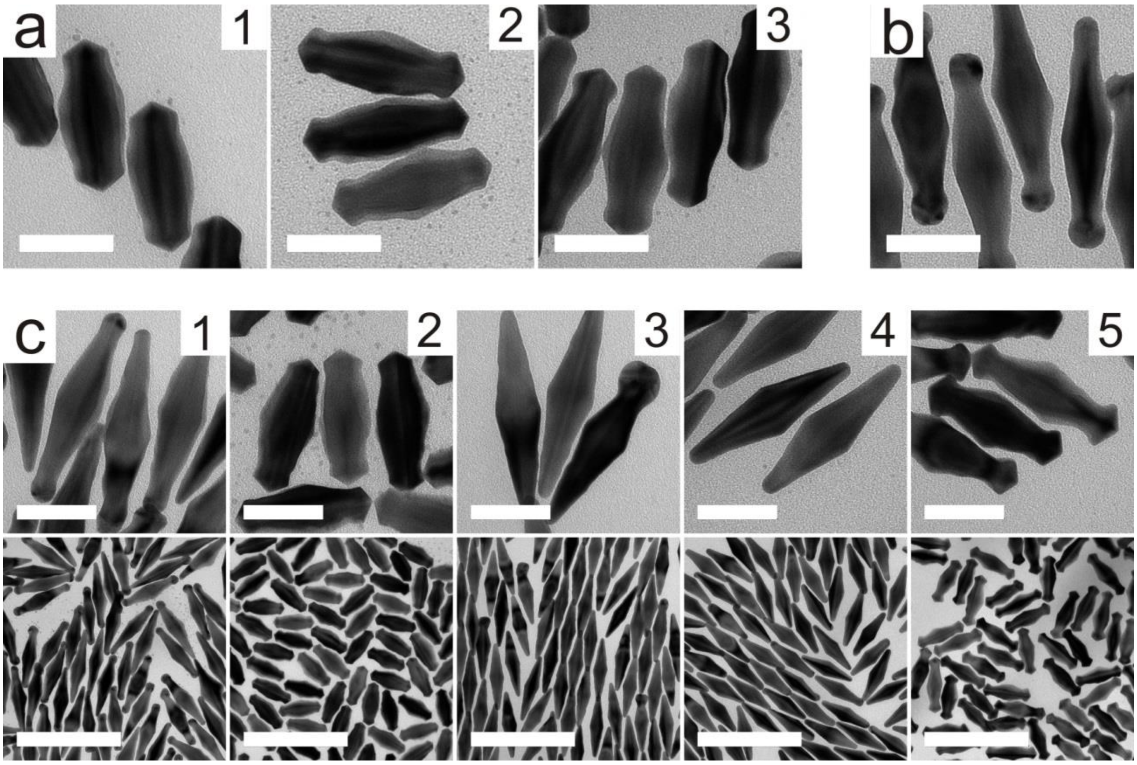

Supplementary Figure 6. **Regrown bipyramids with binary surfactants. a**, TEM images of regrown structures in Figure 3b-7 with different amounts of $AgNO_3$ and with no added HCl. The regrowth solution consists of 2 mM $HAuCl_4$ (50 µL), 20 µL of nanopure water in place of 1 N HCl (20 µL) and 10 mM ascorbic acid (8 µL) with varying concentrations of $AgNO_3$; 10 µL of 1 mM, 2 mM and 4 mM corresponding to images 1 through 3 above, with binary surfactant [CTAC]:[CTAB] = 90:1. **b**, TEM image of regrown structures in Figure 3b-6 with more HCl used. The regrowth conditions are identical to those used in Figure3b-6, except 30 µL of 1 N HCl was used. **C**, TEM images of regrown structures with binary surfactants [CTAC]:[CTAB] = 900:1. The regrowth conditions are identical to those used in Figure 3: specifically, 1 is standard growth solution, 2 has no HCl, 3 has no $AgNO_3$, 4 has no HCl and no $AgNO_3$, and 5 has 5x $AgNO_3$. All scale bars are 200 nm and 50 nm for low and high magnification images, respectively.

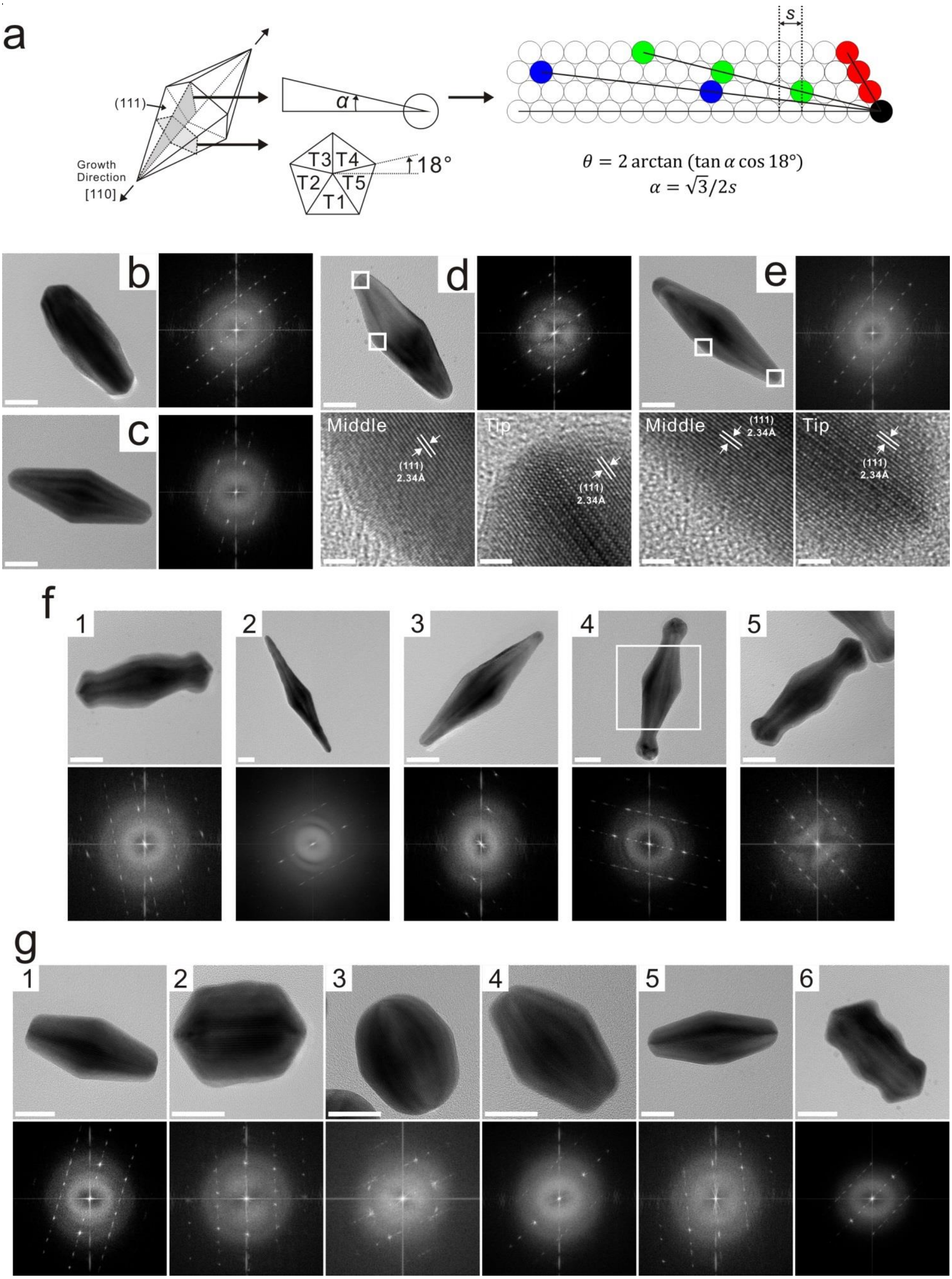
a
(111)
Growth Direction
[110]
α
T3 T4
T2 T5
T1
18°
s
θ = 2 arctan (tan α cos 18°)
α = √3/2s
b
c
d
e
Middle
Tip
(111)
2.34Å
f
1
2
3
4
5
g
1
2
3
4
5
6

Supplementary Figure 7. **HR-TEM images and FFT patterns of regrown structures with singular and binary surfactants. a**, Illustrations for cyclic penta-tetrahedral twinning of bipyramids. Each twinning plane is labelled from T1 to T5. The two gray areas show the cross-section of the bipyramid perpendicular to the growth dirction and inter-plane between T4 and T5. The equation shows the relationship between measured tip angle (θ) of the structures in orientation 1 in Fig. 4a and average step length (s). Regrown bipyramids in Fig. 4c (b), 4d (c), 3a-4 (d), 3a-5 (e), 4e (f-1), 4f (f-2), 4g (f-3), 4h (f-4, FFT shows boxed area), 4i (f-5), 6a-1 (g-1), 6a-2 (g-2), 6a-3 (g-3), 6a-4 (g-4), 6a-5 (g-5) and 6b (g-6), respectively. Magnified images showing the lattice fringes represent the areas of marked white boxesat the tip and middle of particles. All scale bars are 20 nm and 2 nm for low and high magnification images, respectively.

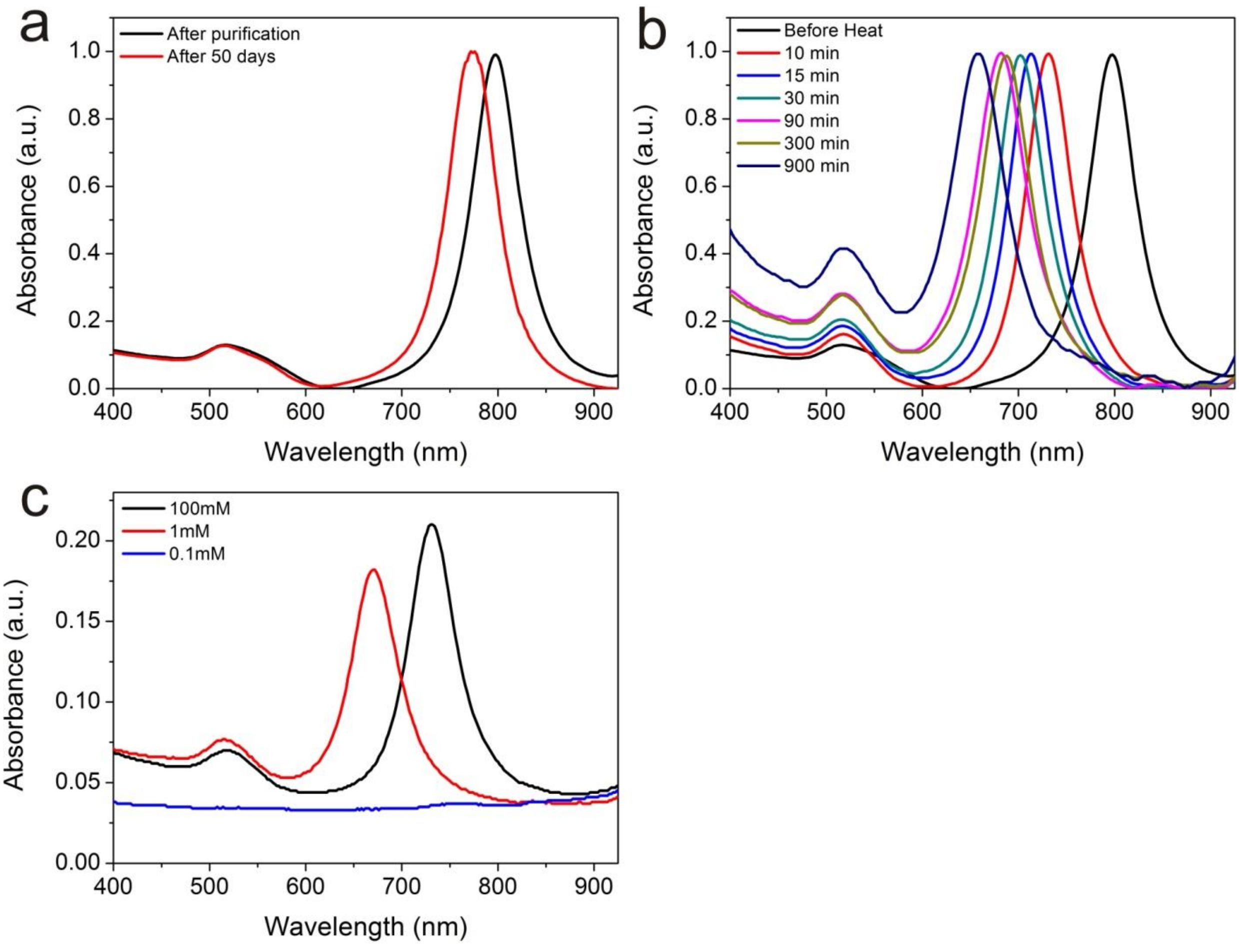

Supplementary Figure 8. **UV-Vis-NIR spectra resulting from oxidative etching. a**, UV-Vis-NIR spectra of bipyramids immediately after purification and after 50 days stored at room temperature. **b**, UV-Vis-NIR spectra from oxidatively etching the bipyramids in the presence of 100 mM CTAB at 120 ºC. After 10, 15, 30, 90, 300 and 900 min, the vials were cooled down with room temperature water to halt the oxidative process. **c**, Spectra from heating for 10 min at 120 ºC with 100 mM, 1 mM, 0.1 mM CTAB. The effect of CTAC was not studied because of the low stability of the bipyramids in the presence of CTAC.

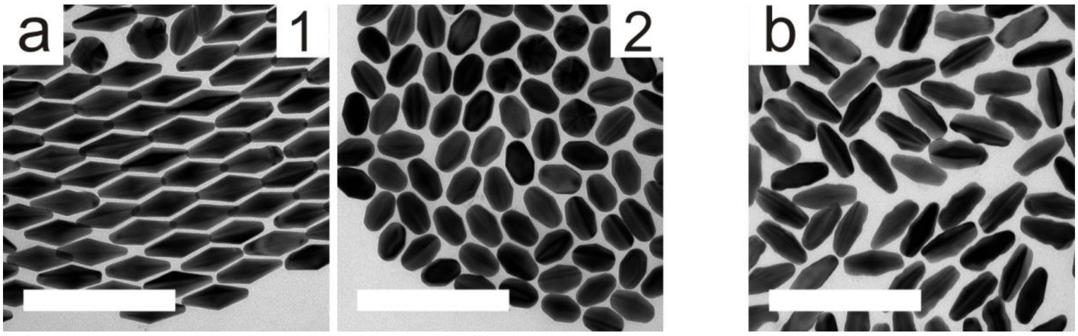

Supplementary Figure 9. **Regrown structures from oxidatively-etched particles. a**, Regrown structures from etched rice-shaped particles in Figure 5a-2 with a standard growth solution (1) and growth solution without $AgNO_3$ (2) with CTAB singular surfactant (See also Supplementary Table 1 for details). **b**, TEM image of regrown structure from etched rod-shaped particle as in Figure 5a-3 with standard growth solution with binary surfactants [CTAC]:[CTAB] = 90:1. All scale bars are 200 nm.

Fig. No.	Surfactant		HAuCl$_4$		AgNO$_3$		AA*		1 N HCl	Vol.(µL) of BP* seeds	pH*	[CTAB]:[CTAC]
	0.1M CTAB Vol.(µL)	0.1M CTAC Vol.(µL)	Conc.(M)	Vol.(µL)	Conc.(M)	Vol.(µL)	Conc.(M)	Vol.(µL)	Vol.(µL)			
2c-6			0.002	50	0.002	10	0.02	8		50 (in 1 mM CTAB)		
2c-7										10 (in 1 mM CTAB)		
2c-8	900	-							20	30 (in 1 mM CTAB)	1.73	CTAB only
2c-9			0.004	50	0.004	10	0.04	8		20 (in 1 mM CTAB)		
2c-10										10 (in 1 mM CTAB)		
S2a-1			0.001		0.001		0.01					
S2a-2			0.004		0.004		0.04			100 (in 1 mM CTAB)		
S2a-3			0.01		0.01		0.1					
S2a-4			0.02		0.02		0.2					
S2a-5			0.002		0.002		0.02		20	5 (in 1 mM CTAB)	1.73	
S2a-6			0.004		0.004		0.04					
S2b-1	900	-		50	0.002	10		8				CTAB only
S2b-2					0.004							
S2b-3					0.008							
S2b-4			0.002				0.02		20 (0.2N)	100 (in 1 mM CTAB)	2.435	
3a-1, S2b-5					0.002				20		1.73	
S2b-6									24		1.656	
S4b-1										100 (in 0.1 mM CTAB)		1:9000
S4b-2										100 (in 1 mM CTAB)		1:900
S4b-3										100 (in 2 mM CTAB)		1:450
3b-6, S4b-4	-	900	0.002	50	0.002	10	0.02	8	20	100 (in 10 mM CTAB)	1.73	1:90
S4b-5												1:9
S4b-6	400	500										1:1
S4b-7	800	100	0.002	50	0.002	10	0.02	8	20	100 (in 100 mM CTAB)		9:1
S4b-8	800	10 (0.01M)	0.002	50	0.002	10	0.02	8	20			90:1
3a-2					0.002	10			20µL DIW added		3.62	
3a-3	900	-	0.002	50			0.02	8	20	100 (in 1 mM CTAB)	1.73	CTAB only
3a-4					10µL DIW added				20µL DIW added		3.62	
3a-5					0.01	10			20		1.73	
3b-7					0.002	10			20µL DIW added		3.62	
3b-8	-	900	0.002	50			0.02	8	20	100 (in 10 mM CTAB)	1.73	90:1
3b-9					10µL DIW added				20µL DIW added		3.62	
3b-10					0.01	10			20		1.73	
6a-1									20		1.73	
6a-2					0.002	10			20µL DIW added		3.62	
6a-3	900	-	0.002	50			0.02	8	20	Rod seeds from etching 100 (in 1 mM CTAB)	1.73	CTAB only
6a-4					10µL DIW added				20µL DIW added		3.62	
6a-5					0.01	10			20		1.73	
6b		900	0.002	50	0.01	10	0.02	8	20	Rod seeds from etching 100 (in 10 mM CTAB)	1.73	90:1
S9a-1	900	-			0.002	10				Rice seeds from etching 100 (in 1 mM CTAB)		CTAB only
S9a-2			0.002	50	10µL DIW added		0.02	8	20		1.73	
S9b	-	900			0.002	10				Rod seeds from etching 100 (in 10 mM CTAB)		90:1

Supplementary Table 1. The regrowth conditions employed with either singular or binary surfactants. Total reaction volume remains constant across all trials (1.088 mL). *AA= Ascorbic Acid, BP= Bipyramid, pH= calculated values.

No.	Length (nm)	Width (nm)	AR	LSPR (nm)
1	68.3 ± 3.6	22.8 ± 1.2	3.0	778
2	74.8 ± 2.9	22.7 ± 1.5	3.3	800
3	77.9 ± 3.0	24.5 ± 1.5	3.2	810
4	80.4 ± 2.8	23.6 ± 1.2	3.4	814
5	94.9 ± 2.9	30.8 ± 1.6	3.1	822
6	113.1 ± 3.9	34.7 ± 1.4	3.3	811
7	141.0 ± 4.8	47.9 ± 1.4	3.0	844
8	172.3 ± 4.3	51.8 ± 2.2	3.3	933
9	202.7 ± 3.2	56.7 ± 2.0	3.6	975
10	239.7 ± 5.0	64.5 ± 3.6	3.7	1054

Supplementary Table 2. The table shows the summary of the lengths and widths, aspect ratios (AR), and longitudinal surface plasmon resonance (LSPR) peaks, corresponding to extinction spectra 1-10 in Figure 1c.

Fig. No.	Length (nm)	STDEV*	PD* (%)	Width (nm)	STDEV*	PD* (%)	AR*	Tip Width (nm)	STDEV*	PD* (%)
**3a-1**	99.5	2.6	2.6	30.2	0.9	3.0	3.3			
**3a-2**	86.4	2.5	2.9	35.9	1.1	3.0	2.4			
**3a-3**	74.5	2.6	3.6	32.5	0.8	2.6	2.3			
**3a-4**	99.8	4.4	4.4	34.7	1.1	3.1	2.9			
**3a-5**	107.6	3.3	3.1	33.4	1.3	3.8	3.2			
**3b-6**	115.5	5.9	5.1	30.0	1.5	5.1	3.8	17.3	2.6	15.2
**3b-7**	84.3	3.5	4.1	30.4	1.6	5.1	2.8	22.3	1.5	6.5
**3b-8**	137.7	7.6	5.5	28.5	1.1	4.0	4.8	11.9	0.9	7.6
**3b-9**	100.8	3.7	3.7	27.1	1.1	4.2	3.7			
**3b-10**	97.7	4.9	4.0	31.1	1.7	5.3	3.1	22.5	1.4	6.4
**5a-2**	58.3	3.2	3.8	23.4	1.2	5.2	2.5			
**5a-3**	46.3	2.1	3.6	22.8	1.0	4.3	2.0			
**6a-1**	76.2	2.5	3.4	33.9	1.1	3.2	2.2			
**6a-2**	52.3	2.4	3.2	39.4	1.9	4.8	1.3			
**6a-3**	52.5	1.9	3.0	39.3	1.0	2.5	1.3			
**6a-4**	60.5	3.3	2.8	38.7	1.6	4.2	1.6			
**6a-5**	82.3	2.7	2.5	33.9	0.9	2.6	2.4			
**6b**	65.7	2.3	2.3	35.7	2.7	7.5	1.8	34.4	2.4	7.0

Supplementary Table 3. Dimensions of regrown structures, both bipyramids and oxidatively-etched nanorods, with singular and binary surfactants. *STDEV= standard deviation, PD= polydispersity, AR= aspect ratio.

Supplementary Note 1. Depletion flocculation as a concept was first proposed by Asakura and Oosawa in 1958[1], and applied as a purification technique for nanoparticles by Park in 2010[2]. The first nanoparticle systems to be purified involved nanorods and nanospheres. Park introduced a model to predict when nanoparticles would begin to flocculate. The simplified equation, shown below in Equation 1, relates the effective micelle concentration and contact area of the nanoparticle to the potential, *U*. When $|U| \approx 4\text{-}5k_BT$, the particles will begin to flocculate out of solution[3].

$$|U| = \frac{(2r_m)A(c - cmc)}{n} N_0 k_B T \qquad \text{(Eq. 1)}$$

In Equation 1 above, r_m is the radius of the surfactant micelle, *A* is the possible contact area of the nanoparticle, *c* is the surfactant concentration, *cmc* is the surfactant's critical micelle concentration, *n* is the aggregation number of the surfacant micelle, N_0 is Avogadro's number, k_B is the Boltzmann constant, and *T* is the temperature. For the purpose of calculating the contact area of the bipyramid, the shape was assumed to be a pentagonal bipyramid with 10 equivalent triangular faces. The constants for the surfactant micelles were all obtained from literature sources.

The reason BDAC was chosen for the depletion flocculation was its higher effective micelle concentration than CTAB. For BDAC, rm=2.4 nm[4], cmc=0.0005 M[5], n=62[6] and for CTAB, rm=3.0 nm[7], cmc=0.001 M[8], n=162[9]. This results in an effective micelle concentration roughly 2.6 times larger, and a potential to flocculate roughly 2.09 times higher (due to the smaller micelle size). The high micelle concentration is necessary because bipyramids, having ten identical faces, actually have a relatively low contact area compared to nanorods of similar size, which essentially have five faces.

The range of purified bipyramids only extends from 68 nm to 95 nm while the range of bipyramids available through the seed-mediated growth extends to sizes both shorter and longer than that. Shorter bipyramids are difficult to purify because the corresponding BDAC concentration necessary to purify is around 400 mM or more. At concentrations this high, the viscosity is too high to allow for the flocculation to occur in a reasonable time frame. For bipyramids synthesized larger than 95 nm, the psuedo-spherical impurities are also proportionally larger. As these impurities grow larger, they become less spherical and more faceted. These facets become large enough that they begin to co-flocculate with the bipyramids, effectively preventing any possible purification based on size selectivity.

Supplementary References

1. Asakura, S. & Oosawa, F. Interaction between particles suspended in solutions of macromolecules. *J. Polym. Sci.* **33,** 183-192 (1958).

2. Park, K., Koerner, H. & Vaia, R. A. Depletion-induced shape and size selection of gold nanoparticles. *Nano Lett.* **10,** 1433-1439 (2010).

3. Leal-Calderon, F. *et al.* Aggregation phenomena in water-in-oil emulsions. *Langmuir* **12,** 872-874 (1996).

4. Rodríguez, J. R. & Czapkiewicz, J. Conductivity and dynamic light scattering studies on homologous alkylbenzyldimethylammonium chlorides in aqueous solutions. *Colloids Surf. A.* **101,** 107-111 (1995).

5. Bakshi, M. & Kaur, I. Head-group-induced structural micellar transitions in mixed cationic surfactants with identical hydrophobic tails. *Colloid. Polym. Sci.* **281,** 10-18 (2003).

6. Alargova, R. G., Kochijashky, I. I. & Zana, R. Fluorescence study of the aggregation behavior of different surfactants in aqueous solutions in the presence and in the absence of gas. *Langmuir* **14,** 1575-1579 (1998).

7. Berr, S. S. Solvent isotope effects on alkytrimethylammonium bromide micelles as a function of alkyl chain length. *J. Phys. Chem.* **91,** 4760-4765 (1987).

8. Li, N., Liu, S. & Luo, H. A new method for the determination of the first and second CMC in CTAB solution by resonance rayleigh scattering technology. *Anal. Lett.* **35**, 1229-1238 (2002).

9. Aswal, V. K. & Goyal, P. S. Role of different counterions and size of micelle in concentration dependence micellar structure of ionic surfactants. *Chem. Phys. Lett.* **368**, 59-65 (2003).
